# The School Age and School Hours

**Published:** 1888-10

**Authors:** 


### THE SCHOOL AGE AND SCHOOL HOURS.

Dr. C. W. Chancellor, of the Maryland board of health, has recently given an interesting summary of those sanitary requirements which relate to our school system and the public education of children.

After taking up the subject of school-house construction and site, the writer discusses the question of the age at which education should begin. He cites a number of authorities, from Aristotle to Hufeland, all tending to show that the school work should not begin until the eighth year. The authority of Spurzheim is quoted in support of the view that "precocity is almost always a disease;" "an envious frost, which nips the blossoms because they appear quickly."

Mental and physical training may begin, as Quintilian recommends, at the third or fourth year. But while our schools are arranged as at present, it may be wise to accept the modern view, that the school age should begin at seven or eight.

Ribot has shown that capacity to fix the attention is a characteristic of more civilized and intellectual people. So it is also of more mature minds. The infant has no such power; the child of three wearies after a few minutes' effort at following some train of thought or memorizing process.

According to Dr. Newell, "for children of ten or twelve years the capacity for bright and voluntary attention is exhausted by four varied lessons requiring mental effort of half an hour each, with intervals of relief in the forenoon. In the afternoon this capacity is reduced one-half. Two hours in the forenoon and one hour in the afternoon, is as long a time as children can be profitably employed in school."

Dr. Chancellor supports this view, and advocates eighteen hours a week as the limit for school children under twelve. Baginskey, author of a German work on school hygiene, takes the same view, and in demonstration of its effectiveness it is alleged that half-time pupils in the English schools learn as much as the children who are in school the full number of hours.

